# Indirect reciprocity can overcome free-rider problems on costly moral assessment

**DOI:** 10.1098/rsbl.2016.0341

**Published:** 2016-07

**Authors:** Tatsuya Sasaki, Isamu Okada, Yutaka Nakai

**Affiliations:** 1 Faculty of Mathematics, University of Vienna, Oskar-Morgenstern-Platz 1, 1090 Vienna, Austria; 2 Department of Business Administration, Soka University, 1-236 Tangi-cho, Hachioji-city, Tokyo 192-8577, Japan; 3 Faculty of Systems Engineering and Science, Shibaura Institute of Technology, Fukasaku 2307, Minuma-ku, Saitama-city, Saitama 337-8570, Japan

**Keywords:** evolution of cooperation, indirect reciprocity, social norm, second-order free-rider, pool punishment, replicator dynamics

## Abstract

Indirect reciprocity is one of the major mechanisms of the evolution of cooperation. Because constant monitoring and accurate evaluation in moral assessments tend to be costly, indirect reciprocity can be exploited by cost evaders. A recent study crucially showed that a cooperative state achieved by indirect reciprocators is easily destabilized by cost evaders in the case with no supportive mechanism. Here, we present a simple and widely applicable solution that considers pre-assessment of cost evaders. In the pre-assessment, those who fail to pay for costly assessment systems are assigned a nasty image that leads to them being rejected by discriminators. We demonstrate that considering the pre-assessment can crucially stabilize reciprocal cooperation for a broad range of indirect reciprocity models. In particular for the most leading social norms, we analyse the conditions under which a prosocial state becomes locally stable.

## 1. Introduction

Natural selection disfavours indirect reciprocity unless the costs of observation and assessment are negligible [1]. According to proper social norms that distinguish good from evil, such as image-scoring norm [2], indirect reciprocity can promote cooperation even in large populations [3]. However, making moral assessments takes time and effort. Discriminators who incur no assessment cost thus appear as free-riders that erode a cooperative state achieved by discriminators who incurs the costs. Despite the advance of indirect reciprocity, the crucial question remains unsolved [4]: how can cooperation through indirect reciprocity be maintained when considering the costs associated with the assessment system?

To address this crucial question, we focus on the fact that this puzzling situation is closely related to the second-order free-rider problem in costly punishment [1]. The evolution of costly punishment, in striking contrast to indirect reciprocity, has been given much more attention over the last decades. In tackling the second-order free-rider problem, previous study significantly examined pool punishment [5–10]. The key aspect of pool punishment is its proactive mechanism to detect the second-order free-riders through unconditional prepayment. The mechanism paves the way for effectively punishing the second-order free-riders.

In this paper, we apply the essence of the pool-punishment mechanism to fix the issue of the costly moral assessment. In the next section, we introduce a basic model of indirect reciprocity and the known negative outcome from considering the assessment costs. In the Results section, we show how adopting a proactive assessment mechanism can improve the outcome.

## 2. Material and methods

We build upon the standard framework for the evolution of indirect reciprocity by reputation [11,12]. Using the framework, a strategy for discriminators is given by an assessment rule combined with an action rule. We base indirect reciprocity on the giving game, which is a two-player game in which one player acts as a donor and the other a recipient. The donor can choose to help the recipient by giving benefits *b* > 0 at personal cost *c* > 0 or not to help. We consider the following implementation error: a player who has intended to help involuntarily fails to do so with a probability *e* [1,13].

We start with a basic model in which each individual is endowed with a binary image score of ‘good’ or ‘bad’. It is assumed that the discriminator's action rule is to help a good recipient or not to help a bad recipient. After observing every giving game, a unique assessment system assigns the donor's image by following a specific assessment rule. We assume that all discriminators share the same list of individual image scores provided by the assessment system. We later consider in particular the second-order assessment rule, which is a function of the donor's last action and the recipient's last image (table 1).

**Table 1.** How the second-order rules make moral assessments in giving games with pre-assessment. ‘G’ and ‘B’ describe a good and bad image, respectively. In the donor's action, ‘C’ and ‘D’ describe giving help and refusing help, respectively.

conditions	recipient's image	G and nice	G and nice	B or nasty	B or nasty
	donor's action	C	D	C	D
assessment rule: what does the donor's image look like?	simple standing	G	B	G	G
	stern judging	G	B	B	G

To study the evolution of discriminators, we consider a continuous-entry model: an individual's birth and death sometimes happen, and this changes the strategy distribution in the population [14]. We assume that in one's lifetime an individual infinitely plays the one-round giving game with different opponents. We consider infinitely large populations and analyse the replicator dynamics [15] for the following four strategies: (i) paying discriminator [*Z*] is willing to help a good recipient and refuses to help a bad recipient in the giving game. Also s/he is willing to pay for the assessment cost *k* > 0. (ii) Evading discriminator [*W*] similarly acts as a paying discriminator in the giving game, except that s/he is not willing to pay for the assessment cost. (iii) Cooperator [*X*] unconditionally intends to help a potential recipient and (iv) defector [*Y*] unconditionally intends not to help a potential recipient. Both cooperator and defector are not willing to pay for the assessment cost. We denote by *x*, *y*, *z* and *w* the frequencies of cooperators, defectors and paying and evading discriminators, respectively. The replicator dynamics for these strategies are described as d*n*/d*t* = *n*(*P_S_* − *P*), where *n* is the frequency of strategy *S* (=*X*, *Y*, *Z*, *W*), *P_S_* is the expected pay-off given by the limit in the mean of the pay-off per round for strategy *S* and *P* is the average pay-off over the population, given by *xP_X_* + *yP_Y_* + *zP_Z_* + *wP_W_*.

To formalize the expected pay-offs, we denote by *g_S_* the probability that a recipient with strategy *S* is helped by a given discriminator. In the basic model, this is identical to the fraction of good players within all *S* strategists. Let *g* be the population average of *g_S_*, thus *g* = *xg_X_* + *yg_Y_* + *zg_Z_* + *wg_W_*. The population size is very large, so we may assume that the population configuration for *g_S_* does not change between the consecutive one-round giving games [16]. Thus, the expected pay-offs are described as2.1
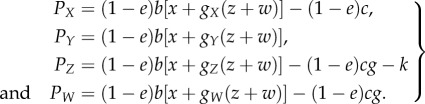
We note that in the basic model either paying or evading discriminators intend to help a potential recipient who has a good image, thus leading to *g_z_* = *g_W_*. This results in paying discriminators being worse off than evading discriminators. Substituting this into equation (2.1) yields2.2
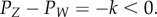


For any degree of the assessment cost *k*, evading discriminators dominate paying discriminators in the interior state space, and thus the population in the end attains a state that excludes paying discriminators. In the absence of cost payers, the assessment system cannot be established. Consequently, cooperation in that case would vanish without discrimination.

## 3. Results

The basic model reveals that considering cost evaders destabilizes indirect reciprocity irrespective of the assessment rule, as shown in previous work [1]. To stabilize indirect reciprocity, we examine an institutional variant of the basic model. As a first step, we extend the basic model to a two-stage game in which one round consists of the stage of payment for the observation costs followed by the stage of the giving game, which is the same as in the basic model. The first stage offers an opportunity to transfer some fees to a central account as in automatic utility payments.

The essential idea is to specifically assess the second-order free-rider. We consider a different binary moral code ‘nice’ or ‘nasty’. The (unique) assessment system assigns a nice image to an individual if s/he pays the costs in the first stage, otherwise that individual is assigned a nasty image. In evaluating the donor's action of the giving game, as the first step we simply apply the existing assessment framework to the second stage, as in the basic model.

We keep the four strategies, cooperators, defectors, paying discriminators and evading discriminators, as before and assume that in the first stage, paying discriminators are willing to pay but the remaining cooperators, defectors and evading discriminators are not. We also modify the discriminator's action rule for the giving game as follows: either paying or evading discriminators give help if a potential recipient has a good *and* nice image, or otherwise (if bad *or* nasty), refuse help.

The extra assessment by the utilities payment system seriously lowers the image score for the second-order free-riders. For analytical simplicity, we assume that the utilities payment system is so perfect that no assessment error occurs for the first stage. All of the evading strategies: cooperators, defectors and evading discriminators (*X*, *Y* and *W*), therefore, are necessarily assessed as nasty. This yields *g_X_* = *g_Y_* = *g_W_* = 0. (Note that with the extra assessment, *g_S_* equals the probability of good and nice players.) Thus, equation (2.1) becomes3.1
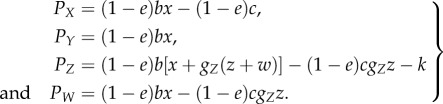


It is clear that *P_Y_* ≥ *P_W_* ≥ *P_X_*. To understand when the homogeneous state of paying discriminators *z* = 1 becomes locally stable, it is enough to check if *P_Z_* − *P_Y_* > 0 in the vicinity of *z* = 1 on the face *x* = *w* = 0. This yields3.2
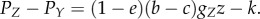


With suitable assessment rules, it is possible to have that *g_Z_* > 0 in the vicinity of *z* = 1. In this case, the node *z* = 1 turns into a locally stable equilibrium when the net benefit *b* − *c* is sufficiently large compared to the assessment cost *k*.

Finally, we demonstrate how the model with the extra assessment improves the results for some of the most leading assessment rules. We examine simple standing [13,16] and stern judging [17], the only two second-order assessment rules in the leading eight norms [10,11]. According to the discriminator's action rule in the variant, we extend simple standing and stern judging as in table 1. These rules assign a good image to those who help a good and nice recipient with no implementation error (probability (1 − *e*)*g*) and also a good image to those who refuse to help a bad or nasty recipient (probability 1 − *g*). By assumption of the image dynamics, the sum of these probabilities should equal *g_Z_*. Considering also *g* = *g_Z_z* then leads to the recursive equation for *g_Z_*, *g_Z_* = (1 − *e*)*g_Z_z* + (1 − *g_Z_z*). This yields *g_Z_* = 1/(1 + *ez*). Hence, the necessary and sufficient condition for the homogeneous state of paying discriminators (*z* = 1) to be locally stable either under the simple standing or stern judging rule is3.3
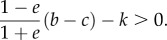


**Figure 1.**
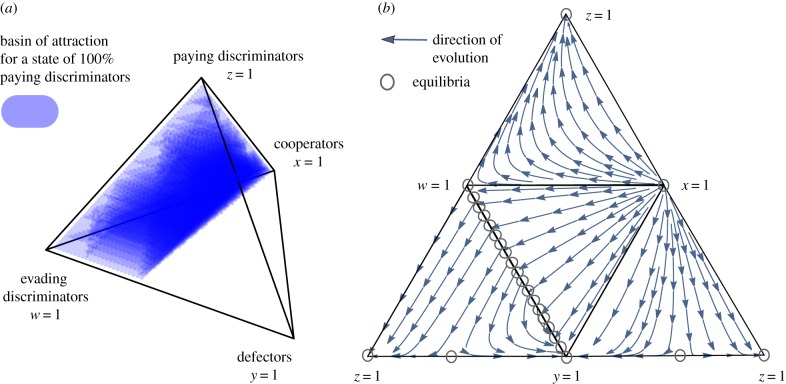
Pre-assessment of cost evaders stabilizes costly indirect reciprocity. (*a*) The tetrahedron describes a simplex of the state space {(*x*, *y*, *z*, *w*): *x* + *y*+*z* + *w* = 1; *x*, *y*, *z*, *w* ≥ 0}. Each corner corresponds to the homogeneous state of each specific strategy. The basin of attraction for paying discriminators covers approximately 61.5% of the whole space. (*b*) The flow diagrams depict the direction of evolution on the boundary faces of the state-space simplex. The state space has no interior equilibrium, and all interior orbits converge to the boundary. Any mixed state of defectors and evading discriminators forms an equilibrium point on the edge *y* + *w* = 1. Parameters: *c* = 1, *b* = 1.5, *e* = 0.01, *k* = 0.3 and simple standing rule. (Online version in colour.)

Figure 1*a* shows the basin of attraction for *z* = 1, and figure 1*b* depicts the flow on the boundary faces of the state space under simple standing. If we assume assessment errors in the first stage, the image dynamics become more complicated but the main results remain qualitatively unchanged—paying discriminators can stabilize with the pre-assessment of cost evaders (electronic supplementary material, S1).

## 4. Discussion

Since the definitive 2013 work by Suzuki & Kimura [1], the evolution of indirect reciprocity relying on costly assessment systems has been explicitly recognized as one of the inevitable issues that challenge the advance of indirect reciprocity [4]. To address the issue, we considered a simple pre-assessment mechanism that is set prior to the primary game in order to detect and label cost evaders. We then demonstrated that the mechanism considered leads to stabilizing costly indirect reciprocity under the most leading social norms, simple standing and stern judging.

Our results are potentially applicable to a broad range of existing indirect reciprocity models, such as tolerant scoring [18], group scoring [19], reputation-based punishment [20], mixed public and private interactions [21], optional interactions [22] and finite populations [23]. On the one hand, managing more complicated assessment systems, such as in [18–21], would be more costly, and thus it is worth considering pre-assessment mechanisms for reducing the temptation to evade cost sharing. On the other hand, as in the case of pool punishment [8], jointly considering optional interactions [22] and finite populations [23] might facilitate establishing pre-assessment mechanisms.

Another promising avenue for future studies would be to explore costly indirect reciprocity on more realistic structured populations. Recent studies using structured populations suggest the importance of cooperator assortment based on reputation [24,25]. However, little is known about how information cost affects reputation-based reciprocity on a network. In the case of the second-order free-rider problem in costly punishment, considering the locality of interactions among players can solve the problem by separating costly punishers from the second-order free-riders [26]. Similarly, the extension to structured populations may lead to significantly different outcomes for paying and evading discriminators.

We left out an advanced issue of analysing non-linking discriminators [27] who act as paying discriminators yet are willing to help cost evaders with a good image. Non-linking discriminators can invade paying discriminators by neutral drift. The preliminary results indicate that considering implementation or assessment errors for the first stage can lead paying discriminators to become better off than non-linking ones, as in fixing neutral drift between conditional and unconditional cooperators [13]. Further investigation is planned in future work.

We note that prepayment for assessment systems can be viewed as a kind of contribution to collective action. Thus, our results corroborate those of previous studies on two-stage games in which reciprocal behaviours in the second stage are linked to a collective action in the first stage. For instance, Panchanathan & Boyd showed that collective action in the first stage can be maintained by considering a shunning strategy that in the second stage withholds help for those who failed to contribute in the first stage [28]. Together, the present results further imply that such a proactive social mechanism, which can discriminate those who deserve to enter social exchange, and reciprocal norms within social exchange may evolve jointly.

## Supplementary Material

Electronic supplementary material for: Indirect reciprocity can overcome free-rider problems on costly moral assessment
