# Supplementary material for: Indirect reciprocity can overcome free-rider problems on costly moral assessment
Source: Biol Lett. 2016 Jul;12(7):20160341. doi: 10.1098/rsbl.2016.0341 (PMC4971172; doi:10.1098/rsbl.2016.0341)
Supplement: Electronic supplementary material for: Indirect reciprocity can overcome free-rider problems on costly moral assessment [file rsbl20160341supp1.pdf]

Electronic supplementary material for:

Indirect reciprocity can overcome free-rider problems on costly moral assessment

Tatsuya Sasaki, Isamu Okada, Yutaka Nakai

S1. Simple standing and stern judging with first-stage assessment errors

We examine assessment errors for the first stage of the variant model considered in the main text. We explore the conditions for the homogeneous state of paying discriminators [Z] with z = 1 to become stable under simple standing and stern judging. We check when paying discriminators become better off than the other three strategies: cooperators, defectors and evading discriminators.

First, we analyse the frequency of good and nice players among *S*-strategists (S = X, Y, Z or W), g_S. As in the main text, we assume that the degree of g_S is unchanged between the consecutive one-round (two-stage) games. We note that by definition the only difference between the rules is with respect to how a potential donor is to be assessed when a potential recipient is bad or nasty and the donor's action is not to help, in which case simple standing assigns a good image and stern judging a bad image.

We denote by e_1 the probability of a first-stage assessment error in which the assessment system involuntarily assesses a paying player (who should have been nice) as nasty or an evading player (who should have been nasty) as nice.

For simple standing, g_S is given by

$$\begin{aligned} g_X &= e_1[(1-e_2)g + (1-g)], \\ g_Y &= e_1[0 \cdot g + (1-g)], \\ g_Z &= (1-e_1)[(1-e_2)g + (1-g)], \\ g_W &= e_1[(1-e_2)g + (1-g)], \end{aligned} \tag{S1}$$

in which g denotes the frequency of players who have both a good and nice image over the whole population, thus $g = xg_X + yg_Y + zg_Z + wg_W$, and e_2 describes the probability of an implementation error in the second stage (see the main text for details). In equation (S1), the

evading players (with X, Y or W) and the paying players (with Z) are assessed as nice with probability e_1 and $1-e_1$, respectively, in the first stage. In addition, the bracket terms of the right side describe the probability that a donor with strategy *S* is assessed as good in the second-stage giving game. When a recipient has a good and nice image (with probability g), X, Z and W strategists are willing to help and are assessed as good with probability $(1-e_2)g$ and Y strategists refuse to help, thus receiving a bad image. Simple standing is a tolerant norm, which is to assign a good image to a donor, irrespective of his/her actions to a recipient who has a bad or nasty image. This leads to the same second term in the bracket as $1-g$ over all g_S.

Then, for stern judging, equation (S1) becomes

$$\begin{aligned} g_X &= e_1[(1-e_2)g + e_2(1-g)], \\ g_Y &= e_1[0\cdot g + (1-g)], \\ g_Z &= (1-e_1)[(1-e_2)g + (1-g)], \\ g_W &= e_1[(1-e_2)g + (1-g)]. \end{aligned} \tag{S2}$$

Stern judging assigns a good image to those who refuse to help a bad or nasty recipient and a bad image to those who help a bad or nasty recipient. This leads to the second term in the bracket for g_X, $e_2(1-g)$, which is the only difference from simple standing in equation (S1).

We analyse the expected payoff P_S at the point $z=1$. Equation (1) in the main text is specified as:

$$\begin{aligned} P_X &= (1-e_2)bg_X - (1-e_2)c, \\ P_Y &= (1-e_2)bg_Y, \\ P_Z &= (1-e_2)bg_Z - (1-e_2)cg - k, \\ P_W &= (1-e_2)bg_W - (1-e_2)cg. \end{aligned} \tag{S3}$$

Considering equations (S1) to (S3), it is obvious that P_W is greater than or equal to P_X. Thus, if $P_Z - P_W\big|_{z=1} > 0$ holds, this yields $P_Z - P_X\big|_{z=1} > 0$.

By solving equations (S1) and (S2) for z = 1, we obtain, in either case of simple standing or stern judging,

$$g\big|_{z=1} = g_Z\big|_{z=1} = \frac{1-e_1}{1+e_2(1-e_1)}. \tag{S4}$$

Substituting equation (S4) into equations (S1) to (S3) yields

$$P_Z - P_Y\big|_{z=1} = \frac{b(1-e_2)[1-(1+e_2)e_1-(1-e_2)e_1^2]-c(1-e_1)(1-e_2)}{1+e_2(1-e_1)} - k, \tag{S5}$$

and

$$P_Z - P_W\big|_{z=1} = \frac{b(1-e_2)(1-2e_1)}{1+e_2(1-e_1)} - k. \tag{S6}$$

Equations (S5) and (S6) (which give the stability threshold conditions) for $z=1$ are common throughout simple standing and stern judging.

As the degree of the first-stage assessment error e_1 goes to 0, equations (S5) and (S6) converge to $\frac{1-e_2}{1+e_2}(b-c)-k$ and $\frac{1-e_2}{1+e_2}b-k$, respectively. Therefore, equation (5) in the main text,

$$\frac{1-e_2}{1+e_2}(b-c)-k>0,$$

is sufficient for $z=1$ to also be stable for a sufficiently small degree of e_1 in either case of simple standing or stern judging.
